# Phenazine virulence factor binding to extracellular DNA is important for *Pseudomonas aeruginosa* biofilm formation

**DOI:** 10.1038/srep08398

**Published:** 2015-02-11

**Authors:** Theerthankar Das, Samuel K. Kutty, Roya Tavallaie, Amaye I. Ibugo, Janjira Panchompoo, Shama Sehar, Leigh Aldous, Amanda W. S. Yeung, Shane R. Thomas, Naresh Kumar, J. Justin Gooding, Mike Manefield

**Affiliations:** 1 School of Biotechnology and Biomolecular Sciences, University of New South Wales (UNSW), Sydney 2052, Australia; 2 School of Chemistry, University of New South Wales (UNSW), Sydney 2052, Australia; 3 Centre for Vascular Research and School of Medical Sciences, University of New South Wales (UNSW), Sydney 2052, Australia

## Abstract

Bacterial resistance to conventional antibiotics necessitates the identification of novel leads for infection control. Interference with extracellular phenomena, such as quorum sensing, extracellular DNA integrity and redox active metabolite release, represents a new frontier to control human pathogens such as *Pseudomonas aeruginosa* and hence reduce mortality. Here we reveal that the extracellular redox active virulence factor pyocyanin produced by *P. aeruginosa* binds directly to the deoxyribose-phosphate backbone of DNA and intercalates with DNA nitrogenous base pair regions. Binding results in local perturbations of the DNA double helix structure and enhanced electron transfer along the nucleic acid polymer. Pyocyanin binding to DNA also increases DNA solution viscosity. In contrast, antioxidants interacting with DNA and pyocyanin decrease DNA solution viscosity. Biofilms deficient in pyocyanin production and biofilms lacking extracellular DNA show similar architecture indicating the interaction is important in *P. aeruginosa* biofilm formation.

With the rapid emergence of resistance to conventional antibiotic therapies, there exists a need to identify new targets for the control of bacterial infection. Such leads are emerging as our understanding of extracellular biofilm matrices and secondary metabolite chemistry improves^1^. Major advances in bacterial biofilm biology have highlighted intercellular signaling (quorum sensing), release of extracellular DNA (eDNA) and production of electrochemically active metabolites (electron shuttles) as key behaviors dictating progression of infection^1^,^2^,^3^. Alongside traditional intracellular targets such as cell wall synthesis or ribosome function, these extracellular phenomena are increasingly perceived as attractive targets for infection control.

*Pseudomonas aeruginosa* is an opportunistic pathogenic bacterium that can cause life-threatening infections in humans such as chronic lung infections, burn wound infections, urinary tract infections and implant or biomaterial associated infections^2^,^3^,^4^,^5^,^6^. eDNA plays a central role in biofilm formation^7^,^8^,^9^ in infections by enhancing surface adhesion^10^,^11^ and cellular aggregation^12^,^13^,^14^, ultimately leading to increased biofilm integrity or strength^9^,^10^,^15^. Redox cycling of pyocyanin generates reactive oxygen species (ROS) that kill host and pathogen cells resulting in eDNA release^16^,^17^. Pyocyanin is also thought to aid respiration in *P. aeruginosa* biofilms by shuttling electrons through biofilm matrices where electron acceptors (oxygen or nitrate) are diffusion limited^1^. Cystic fibrosis patients with *P. aeruginosa* infections have highly viscous sputum with eDNA concentrations of 3–14 mg/ml and pyocyanin concentrations up to 27 μg/ml (~130 μM)^18^,^19^. Recently, pyocyanin was shown to facilitate eDNA binding to *P. aeruginosa* cell surfaces to affect cellular aggregation^20^. While this implies a direct interaction between DNA and pyocyanin, to date, this hypothesis has not been presented or tested.

Considering the significant role of pyocyanin and eDNA in the formation of *P. aeruginosa* biofilms and their relevance to infection, this study sought to elucidate the nature of the potential interaction, consequences for DNA viscosity and potential treatment strategies. Results suggest that pyocyanin preferentially binds to double-stranded (ds) DNA by interacting with the sugar-phosphate backbone and intercalation between base pairs. DNA-pyocyanin intercalation significantly increased the viscosity of DNA solutions and enhanced electron transfer through DNA. Further, the DNA-pyocyanin interaction is disrupted by antioxidants such as ascorbic acid or glutathione. The DNA-pyocyanin interaction is clearly a major molecular feature of *P. aeruginosa* biofilm behavior.

## Results

### Pyocyanin binds dsDNA directly and perturbs its structure

**Figure 1:**
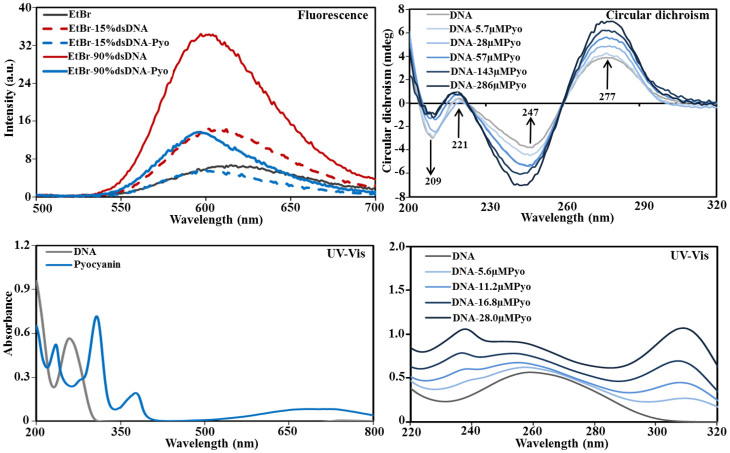
Spectroscopy techniques showing binding of pyocyanin to DNA. Fluorescence emission spectra showing ethidium bromide (4 μM) before and after addition of 15% and 90% dsDNA and the decrease in the intensity of the emission spectra of the ethidium bromide-DNA complex in the presence and absence of pyocyanin (140 μM) (top left). An example of circular dichroism spectra of pure DNA and DNA-pyocyanin (Pyo) complexes in Milli-Q water recorded at 25°C is shown. The change in mdeg of DNA spectra at different wavelengths is due to binding with increasing concentration of pyocyanin (top right). UV-visible spectra of pure DNA and pyocyanin showing a single DNA peak at 259 nm and multiple pyocyanin peaks at 235, 307, 377 and 710 nm (bottom left). Gradual increase in absorbance intensity of the DNA peak and shift of DNA peak from 259 nm to shorter wavelengths with the addition of increasing concentration of pyocyanin (5.6, 11.2, 16.8 and 28.0 μM) in DNA-pyocyanin complex spectra (bottom right).

Figure 1 shows the impact of pyocyanin addition on the fluorescence emission spectra of ethidium bromide (EtBr, 4 μM) bound DNA (6 ng/μl). Two different dsDNA:ssDNA ratios (15:85 and 90:10) were tested. At the lower dsDNA:ssDNA ratio, addition of 140 μM pyocyanin reduced the EtBr peak maxima to that of an EtBr solution without DNA. At the higher dsDNA:ssDNA ratio, pyocyanin addition reduced the peak intensity of the EtBr-dsDNA complex to 40% of its maxima. These data suggest that pyocyanin can displace EtBr from DNA.

Circular dichroism (CD) spectra of DNA-pyocyanin mixtures confirm that DNA binds to pyocyanin with statistically significant changes in peak intensity (*P* < 0.05) at all four characteristic DNA peaks (209, 221, 247 and 277 nm) achieved with pyocyanin concentrations above 28 μM. Additionally, the DNA peak at 247 nm shifted to 244, 243 and 242 nm in the presence of 57, 143 and 286 μM pyocyanin, respectively (Fig. 1). The CD data suggest that pyocyanin creates local perturbations in the DNA double helix structure but does not cause transition in form (B-DNA to A or Z form) and is hence unlikely to affect intracellular DNA function.

Figure 1 also shows the UV-visible range spectra of dsDNA (50 ng/μl) in the presence of pyocyanin (5.6, 11.2, 16.8 or 28.0 μM). The spectra of DNA with pyocyanin showed a gradual increase in absorbance intensity of the DNA peak and a shift of the DNA peak from 259 nm to 253 nm with increasing pyocyanin concentration. The observed hypsochromic effects are indicative of the intercalation of pyocyanin between base pairs and exposure of nitrogenous base pairs due to unwinding of the DNA helix^21^.

**Figure 2:**
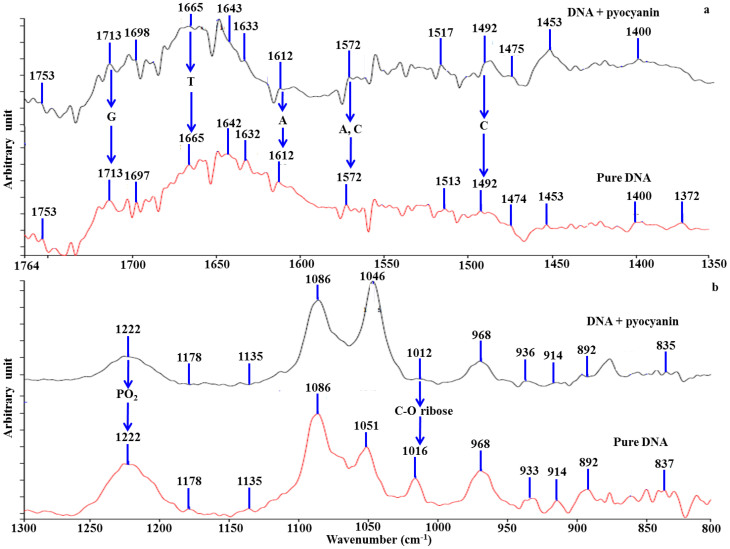
FTIR study on DNA-pyocyanin binding. ATR-IR spectra of pure DNA and a pyocyanin-DNA mixture are shown. The addition of pyocyanin to DNA solutions led to a major change in intensity or shift of the peaks at 1665, 1572 and 1492 cm^−1^ for thymine (T), adenine (A) and cytosine (C) respectively (a). Pyocyanin interaction with DNA phosphate (PO_2_^−^) and ribose backbone are demonstrated by the change in intensity or shift of the peaks at 1178, 1135, 1016, 914 and 837 cm^−1^. The B-conformation of DNA was not changed by addition of pyocyanin as indicated by the absence of significant changes at peaks 1222 and 1086 cm^−1^ (b).

ATR-IR spectra from DNA alone or in combination with pyocyanin confirm that binding does not change the form of the helix (PO_2_^−^ and ribose peaks unchanged at 1222 and 1086 cm^−1^; Fig. 2)^22^,^23^. The addition of pyocyanin to DNA led to changes in intensity or shifts of ATR-IR spectra peaks at 1665, 1572 and 1492 cm^−1^ for thymine (T), adenine (A) and cytosine (C)^22^,^23^ respectively (Fig. 2), indicating that the binding is not nucleotide-specific. Pyocyanin interaction with the DNA phosphate (PO_2_^−^) ribose backbone is apparent from the change in intensity or shift of the peaks at 1178, 1135, 1016, 914 and 837 cm^−1^ (Fig. 2).

**Figure 3:**
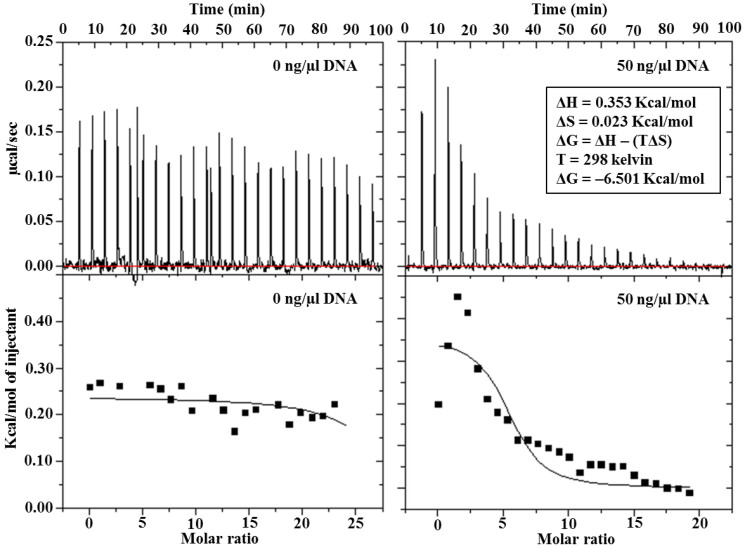
Thermodynamic of DNA-pyocyanin binding. Isothermal titration calorimetry (iTC) studies to evaluate the binding between DNA and pyocyanin. Upper panel: Raw data for the titration of total 200 μl DNA (50 ng/μl) with total ~310 μM pyocyanin. Lower panel: Integrated, dilution-corrected and concentration-normalized titration data of the DNA with pyocyanin. Data were fitted with the ‘one binding site' model using Origin 7.0 data analysis software (MicroCal) with derived thermodynamic parameters including enthalpy (ΔH), entropy (ΔS) and Gibbs free energy (ΔG) at 25°C.

Figure 3 shows isothermal calorimetry analysis of the DNA-pyocyanin interaction. Using a ‘one binding site' model, the overall change in Gibbs free energy (ΔG) for the interaction was calculated to be −6.501 kcal/mol (favourable binding). In comparison, the anti-cancer drug Amsacrine, which binds to DNA via intercalation, has a Gibbs free energy of −5.34 Kcal/mol^24^.

### Pyocyanin facilitates electron transfer mediated by DNA

**Figure 4:**
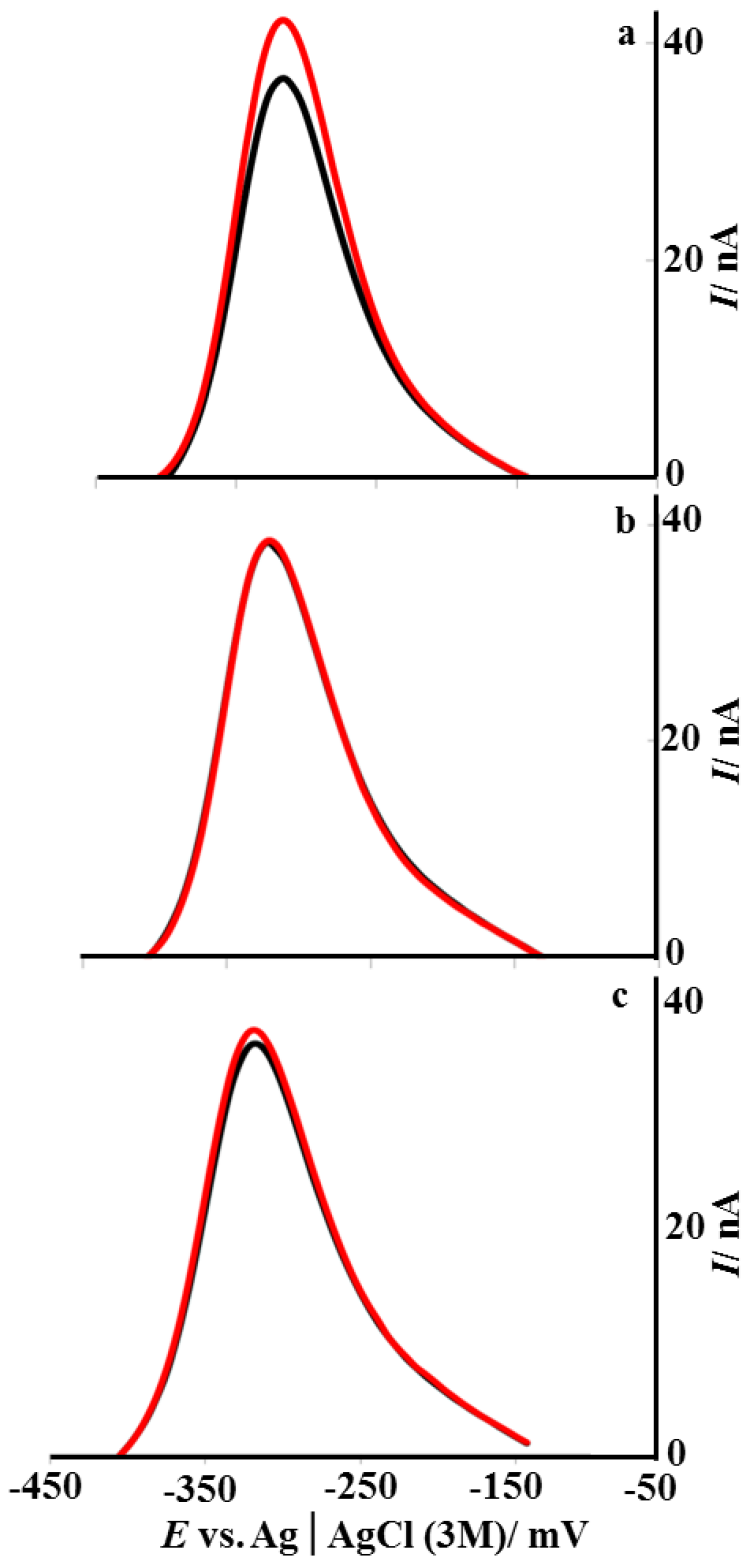
Square wave voltammograms (SWV) of accumulated pyocyanin on modified gold electrodes before (black) and after (red) hybridization with target (a), noncomplementary (b) and single adenine-cytosine mismatch (c). Pyocyanin was accumulated for 30 min in a solution containing 100 μM of pyocyanin in PBS following by rinsing with 0.5 mL PBS. Measurements were performed in a solution containing 2.5 μM pyocyanin in PBS. Pulse amplitude: 25 mV, Step: 4 mV, Frequency: 10 Hz.

Various DNA binding agents including small molecules and proteins influence electron transfer in DNA^25^. Figure 4 illustrates the square wave voltammograms of pyocyanin at electrodes modified with probe DNA before and after hybridization with target, noncomplementary and single base pair mismatch (incorporated mid-sequence) DNA. Compared to ssDNA-modified electrodes, an increase in the square wave voltammetry peak current was observed after hybridization (Fig. 4). The relative change in current caused by hybridization (α) was calculated by dividing the difference between the peak currents before (I_ss_) and after (I_ds_) hybridization by the current before hybridization (I_ss_) using equation (1): 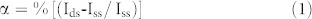
The relative current increase after hybridization with target diminished from 14.6% to 3.3% and 0.3% after hybridization with single base pair mismatch and noncomplementary sequences respectively. These data support a direct interaction between pyocyanin and DNA and reveal that pyocyanin increases electron transfer along the DNA molecule. This may have important implications for respiration by *P. aeruginosa* in biofilms laden with eDNA and pyocyanin representing an undiscovered means of maintaining cellular redox homeostasis by shuttling electrons from anoxic to well aerated regions of biofilms^26^.

### Pyocyanin-mediated increases in DNA viscosity

**Figure 5:**
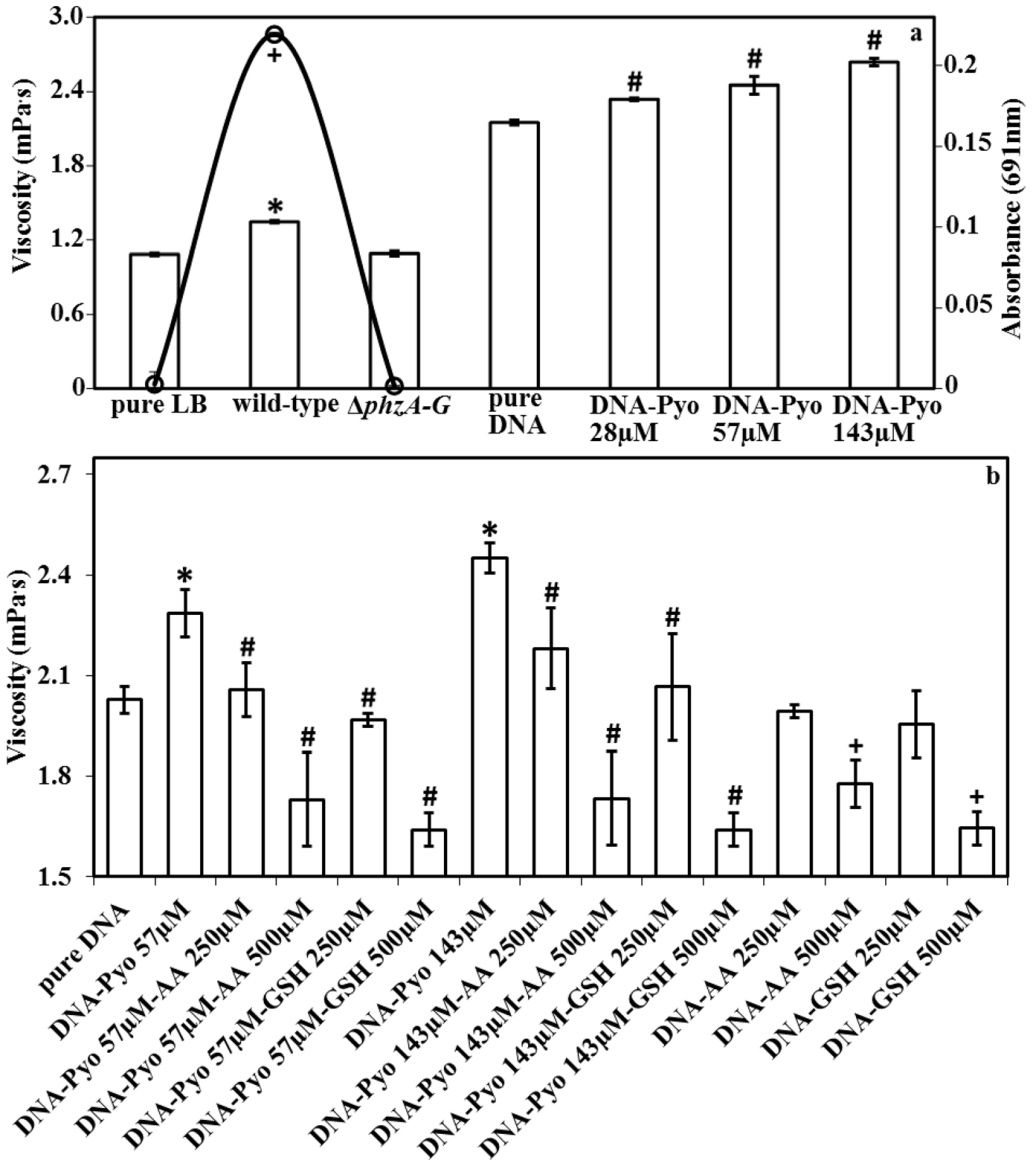
Change in viscosity of DNA solution as a function of pyocyanin, ascorbic acid or glutathione concentration and viscosity of *P. aeruginosa* PA14 biofilm supernatants. Increase in viscosity of a DNA solution with increasing pyocyanin concentration (a). *P. aeruginosa* PA14 wild-type biofilm supernatants show significant increases in viscosity compared with a Δ*phzA-G* mutant (phenazine-deficient) or fresh LB medium (a). The absorbance of biofilm supernatants at 691 nm (pyocyanin absorbance maxima) for fresh LB, wild-type and Δ*phzA-G* after 24 h incubation illustrates pyocyanin production by the wild-type (a). Pyocyanin in the presence of ascorbic acid (AA) or glutathione (GSH) at 250 and 500 μM significantly decreases DNA viscosity. Note that in the presence of 500 μM ascorbic acid or glutathione, the viscosity of the DNA-pyocyanin mixture and DNA solution by itself is lower than the original viscosity of DNA (~2.1 mPa s) at 1.7 and 1.6 mPa s respectively (b). Error bars represent standard deviation from the mean (n = 4). Asterisk, hash and plus symbols indicate statistically significant (*P* < 0.05) differences in comparison to pure LB and Δ*phzA-G* and pure DNA and absorbance, respectively (a). Asterisk, hash and plus symbols indicate that the changes are statistically significant (*P* < 0.05) in comparison to pure DNA, DNA-pyocyanin mixtures and pure DNA, respectively (b).

The high viscosity of sputum from lungs of cystic fibrosis patients has been attributed to eDNA^18^. The viscosity of a 135 ng/μl DNA solution at 20°C increased significantly from 2.1 mPa·s without pyocyanin to 2.3, 2.5 and 2.7 mPa·s with the addition of pyocyanin at 28, 57 and 143 μM respectively (Fig. 5a). The viscosity of pyocyanin in water at 143 μM is identical to that of water at 20°C (1.007 mPa·s). Further, figure 5a illustrates that a phenazine-deficient mutant (Δ*phzA-G*) of *P. aeruginosa* generates supernatants with reduced viscosity. These data suggest that pyocyanin binding to DNA increases solution viscosity. Figure 5b reports the impact of antioxidants glutathione and ascorbic acid on solution viscosity, with a clear decrease evident, ultimately to levels lower than before pyocyanin addition. This data suggests the antioxidants target DNA, pyocyanin or both rather than the mode of interaction between DNA and pyocyanin *per se*.

### Antioxidants interact with DNA and pyocyanin precluding their interaction

**Figure 6:**
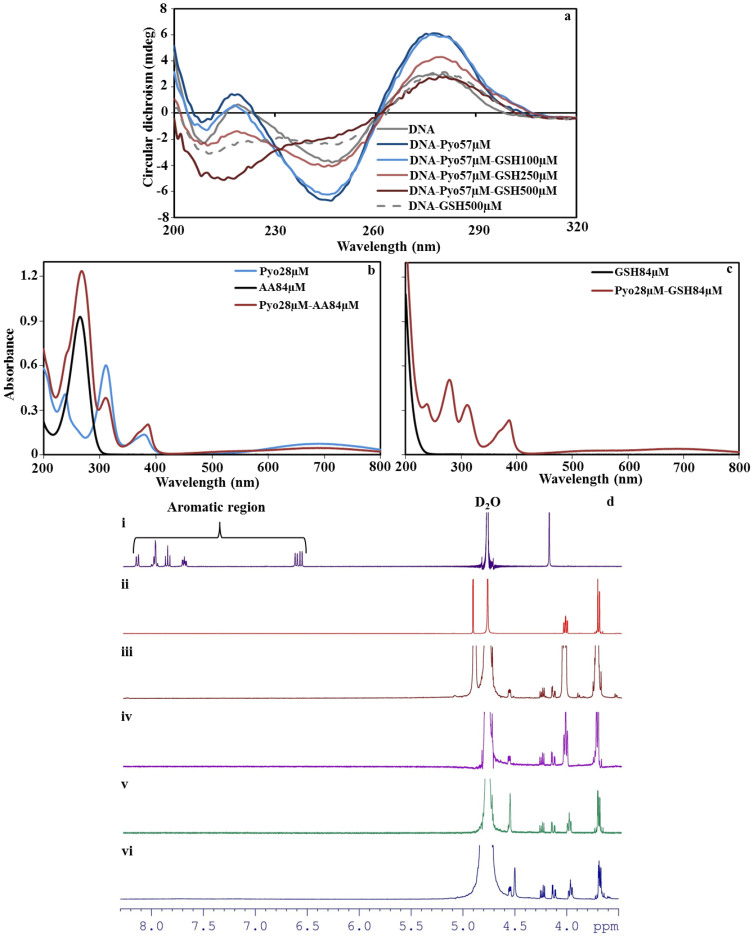
Circular dichroism spectra of pure DNA and a DNA-pyocyanin mixture in the presence and absence of glutathione, and UV-Vis spectra of pyocyanin in the presence and absence of ascorbic acid or glutathione. CD spectra of pyocyanin-DNA demonstrate that as the concentration of glutathione (GSH) increases the intercalation property of pyocyanin (Pyo) decreases. The DNA-pyocyanin intercalation is not affected in presence of 100 μM glutathione, however, as the concentration of glutathione increased to 250 and 500 μM, the pyocyanin intercalation decreased gradually and was inhibited completely at 500 μM. CD spectra of DNA-GSH also confirm a direct interaction between the antioxidant and DNA (a). The UV-Vis spectra of pure pyocyanin, ascorbic acid (AA) and a pyocyanin-ascorbic acid mixture (b) and the pure glutathione and pyocyanin-glutathione mixture (c). The absorbance peak of pyocyanin at 379 nm shifted to 386 nm in the presence of ascorbic acid or glutathione and other pyocyanin absorbance peaks (238 and 311 nm) display altered intensity (b and c). Proton NMR spectra of pyocyanin (i), ascorbic acid (ii) and the pyocyanin-ascorbic acid reaction at ratios of 1:20 (iii), 1:5 (iv), 1:1 (v) and 1:0.5 (vi) at 22°C. The NMR spectra of pyocyanin-ascorbic acid mixtures indicated the disappearance of the pyocyanin aromatic peaks even at low concentration ratios of pyocyanin:ascorbic acid (1:1 and 1:0.5) (d).

Antioxidants glutathione and ascorbic acid are known to disrupt pyocyanin-mediated virulence by interrupting ROS production^27^,^28^. However, in airway epithelial cells, pyocyanin is known to deplete glutathione levels by oxidation^29^. Figure 6a shows CD spectra of DNA-pyocyanin and DNA-pyocyanin-glutathione mixtures. The DNA-pyocyanin intercalation, represented by a peak at 277 nm, is not affected by 100 μM glutathione but decreased with 250 and 500 μM. CD spectra of DNA and glutathione mixtures in the absence pyocyanin however also confirm a direct interaction between the antioxidant and DNA. Antioxidants are known to cause oxidative damage to polymers like DNA, proteins and lipids by releasing free oxygen radicals^30^. Ascorbic acid absorbs strongly in the region of the DNA absorbance maximum so CD spectra of DNA in the presence of ascorbic acid were not obtained.

Ascorbic acid and glutathione alter the color of pyocyanin in solution and affect its absorbance of ultraviolet light. The absorbance peak of pyocyanin at 379 nm shifted to 386 nm in the presence of ascorbic acid or glutathione and other pyocyanin absorbance peaks (238 and 311 nm) displayed altered intensity (Fig. 6b and c).

Proton NMR and EPR have previously been carried out on pyocyanin-glutathione reaction mixtures^27^,^29^, however this has not been performed for ascorbic acid. The NMR spectra of pyocyanin-ascorbic acid mixtures indicated the disappearance of the pyocyanin aromatic peaks even at low concentration ratios (pyocyanin:ascorbic acid 1:1 and 1:0.5; Fig. 6d). In the case of EPR spectra, similar hyperfine coupling patterns were observed in previous reports for pyocyanin, indicating the formation of cation-free radicals^31^ (Sup. Fig. 1). These observations indicated the presence of a non-covalent interaction between the two species and could be due to the protonation and free radical formation of pyocyanin followed by formation of a charge transfer complex between pyocyanin and ascorbic acid^32^. In the charge transfer complexes of pyocyanin and ascorbic acid, pyocyanin will typically act as an electron acceptor while ascorbic acid acts as an electron donor, consistent with the latter's important role as an aqueous physiological antioxidant or reductant^32^. Additionally, the nature of the charge transfer complex would depend on the concentration of ascorbic acid, which is indicated by changes in the color of the mixture at different concentrations (Sup. Fig. 1) and a concentration-dependent up-field shift of the ascorbic acid CH peak in the NMR spectrum. The interaction of pyocyanin with ascorbic acid reported here is distinct from that with glutathione. Pyocyanin reacts with glutathione, leading to formation of a covalent bond between the SH group of glutathione and the pyocyanin aromatic ring^27^.

### Effect of DNase I and pyocyanin on biofilm formation by *P. aeruginosa* PA14 wild-type and a *ΔphzA-G*

**Figure 7:**
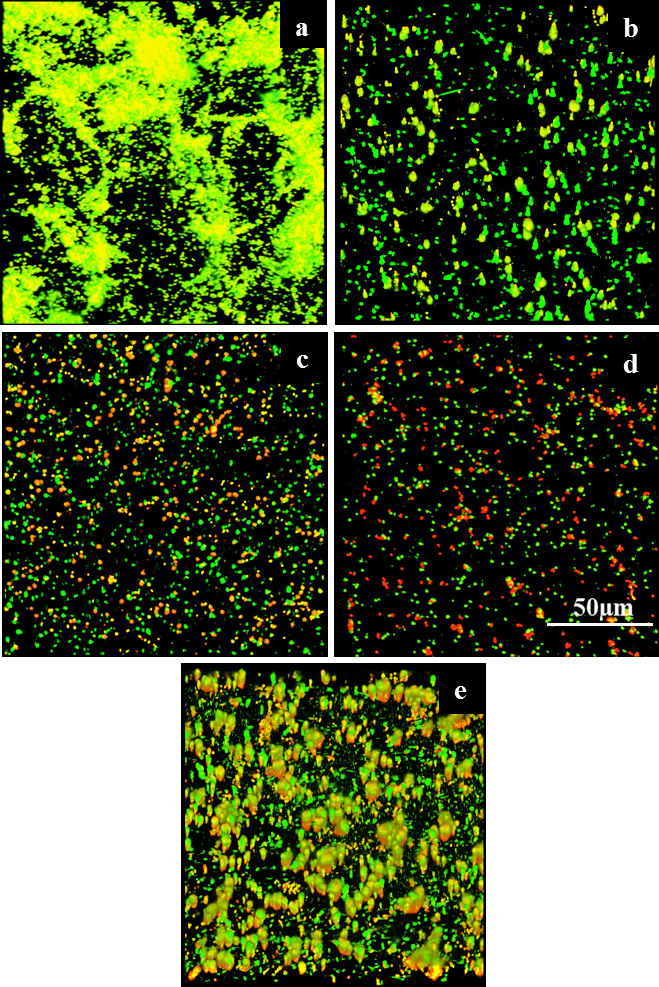
Effect of DNase I and pyocyanin on biofilm formation by *P. aeruginosa* PA14 wild-type and a *ΔphzA-G*. *P. aeruginosa* wild-type and phenazine/pyocyanin deficient mutant (*ΔphzA-G*) biofilm grown under different conditions at 30°C, 150 rpm in LB medium over 24 h.Wild-type (a), wild-type grown in presence of DNase I (b), Δ*phzA-G* mutant (c), Δ*phzA-G* mutant grown in presence of DNase I (d), Δ*phz* mutant grown in presence of 200 μM pyocyanin (e).</emph>

Figure 7 shows confocal laser scanning microscopy images of live/dead-stained *P. aeruginosa* PA14 wild-type and Δ*phzA-G* mutant biofilms grown on a polystyrene substratum over 24 h in the presence or absence of DNase I or pyocyanin. The wild-type strain forms robust biofilms and has significantly higher percentage surface coverage (65 ± 10) in comparison to the wild-type grown in the presence of DNase I and in comparison to the mutant strain (regardless of DNase I) which showed a similar level of percentage surface coverage (23 ± 6). Exogenous addition of pyocyanin to the mutant resulted in an increase in biofilm formation and percentage surface coverage (37 ± 7), although not to the level observed in the wild-type biofilm.

## Discussion

This study reveals that the phenazine pyocyanin, produced by the opportunistic human pathogen *P. aeruginosa*, binds to DNA, resulting in increased electron transfer and structural perturbations that result in increased DNA solution viscosity. *P. aeruginosa* has been comprehensively investigated with respect to its pathogenicity, metabolite production, biofilm formation and eDNA release^1^,^2^,^3^,^4^,^7^,^9^,^33^. Despite the wealth of knowledge, it has gone unnoticed that the virulence factor pyocyanin interacts directly with DNA, affecting properties linked to infection progression. The viscosity of sputum from patients with *P. aeruginosa* lung infections is a determinant of patient outcomes^34^. DNA release by host and pathogen cell lysis is responsible for increasing sputum viscosity and treatments involving enzymatic DNA digestion by inhalation of aerosol DNase have been developed^34^. The discovery that pyocyanin binds to DNA and increases solution viscosity therefore unveils a novel molecular interaction that can be used to target and control *P. aeruginosa*-mediated biofilm formation and its related lung infection.

The discovery that pyocyanin enhances DNA-mediated charge transport is consistent with the use of structurally related molecules (e.g., methylene blue, redmond red) as probes in DNA charge transport studies^25^. The interaction between pyocyanin and DNA presented here is the first such example of enhanced DNA charge transport in a microbiological context. It has previously been proposed that *P. aeruginosa* exploits redox-active metabolites such as pyocyanin to sustain cell viability in biofilms where direct access to electron acceptors such as oxygen or nitrate are diffusion limited^1^. The ability to transfer electrons through biofilm matrices may enable cells to maintain a basal rate of respiration for energy harvesting and to maintain cytoplasmic redox homeostasis^1^, though more work is needed in this area. Significant decreases in biofilm formation by the *P. aeruginosa* wild-type (in presence of DNase I) and the *ΔphzA-G* mutant (regardless of DNase I) and enhancement in *ΔphzA-G* biofilms in the presence of exogenous pyocyanin proves the relevance of DNA-pyocyanin binding for development of *P. aeruginosa* biofilms. Disruption of the pyocyanin-DNA interaction therefore represents a promising target for reducing both the *P. aeruginosa* biofilm and the viscosity of *P. aeruginosa* biofilm exudates in infection settings. Decreases in the viscoelasticity of biofilms or viscosity of sputum in cystic fibrosis patients are known to enhance the performance of antibiotics and other clinical therapies^35^,^36^.

Glutathione concentrations ranging between 250–800 μM are present within the extracellular lung fluid (ELF) representing an important antioxidant defense against pyocyanin-mediated ROS production and subsequent airway epithelial cell damage^27^,^29^. However, evidence exists that cystic fibrosis patients are subjected to increased oxidative stress mediated by pyocyanin that depletes glutathione and ascorbic acid levels in ELF^29^,^37^. A decrease in glutathione levels in the ELF weakens the host immune system^38^. Thus, elevating antioxidant levels (i.e., glutathione and ascorbic acid) within the ELF of cystic fibrosis patients may play a protective role by reducing pyocyanin activity related to ROS production and eDNA interactions.

In conclusion, the data presented here shows that pyocyanin interacts with DNA to elevate DNA viscosity and the DNA-pyocyanin interaction is important for *P. aeruginosa* biofilm formation. This suggests that by inhibiting DNA-pyocyanin intercalation in biofilm matrices, or alteration of one or both players precluding the interaction as observed with antioxidants, biofilm formation and related infections can be impaired.

## Methods

### Characterisation of the DNA-pyocyanin interaction

Calf thymus DNA sodium salt (type I fibres, 42% GC content, Sigma-Aldrich) was dissolved in Milli-Q water. Single-stranded DNA was prepared by sonication. The concentration of double-stranded (ds) DNA present in the DNA stock solution was quantified using a fluorescent dye assay (Qubit, Invitrogen). Stocks of pyocyanin from *P. aeruginosa* (Sigma-Aldrich) were also prepared in Milli-Q water.

Binding of pyocyanin to DNA was assessed with an ethidium bromide-DNA displacement technique^39^, using a Varian Cary Eclipse Fluorescence Spectrophotometer. Double-stranded DNA at 15% or 90% of 6 ng/μl was mixed with ethidium bromide (4 μM) and pyocyanin (140 μM) in SHE buffer (2 mM HEPES, 10 μM EDTA and 9.4 mM NaCl in Milli-Q water adjusted to pH 7 with NaOH). Light emission at 615 nm (λ_ex_ = 480 nm) was quantified at room temperature in a 1-ml quartz cuvette.

UV-Vis spectroscopic scans from 200–800 nm were performed using a Varian Cary 100 Bio UV-Visible spectrophotometer in a 1-ml quartz cuvette on dsDNA (~90%), pyocyanin, the DNA-pyocyanin complex, ascorbic acid, glutathione and pyocyanin-ascorbic acid or pyocyanin-glutathione mixtures in Milli-Q water.

A Chirascan CD spectrophotometer (Applied Photophysics) was used to investigate DNA-pyocyanin, DNA-pyocyanin-glutathione and DNA-glutathione reactions in a 1-mm path length quartz cuvette. Mixtures of dsDNA (~90%) at 135 ng/μl with varying pyocyanin (0, 5.7, 28, 57, 143 and 286 μM) and glutathione (100, 250 and 500 μM) concentrations in 350 μl Milli-Q water were scanned from 200–320 nm after a 15-min static incubation at 25°C.

ATR-Fourier transform infrared (FTIR) spectra were obtained with a Perkin Elmer spectrum 100 FT-IR spectrometer on a Universal ATR sampling accessory at room temperature. One hundred scans were accumulated for each sample in the 4000–650 cm^−1^ spectral range with a resolution of 4 cm^−1^. Background spectra were collected before each measurement. A good subtraction was characterized by a flat baseline around 2200 cm^−1^ caused by the cancellation of the water combination mode (1600 + 600 cm^−1^).

Gibbs free energy (ΔG), enthalpy changes (ΔH), entropy changes (ΔS) and binding stoichiometry (n) of the interaction between DNA and pyocyanin were determined by isothermal calorimetry using a MicroCal iTC 200. dsDNA at 50 ng/μl in Milli-Q water was mixed with pyocyanin (310 μM) and divided into 25 injections, with an interval of 180 sec after each injection. All measurements were performed at 25°C, with a reference power of 5 μcal/sec and stirring speed of 800 rpm. Gibbs free energy changes (ΔG) were determined using the relationship:
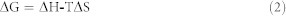
where ΔH is the enthalpy change, T is temperature (Kelvin) and ΔS is the change in entropy.

Nuclear magnetic resonance (NMR) spectroscopy was used to characterize the reaction between pyocyanin and ascorbic acid. NMR spectra were obtained on a Bruker Avance 400 spectrometer from pyocyanin:ascorbic acid mixtures at 1:0.5, 1:1, 1:5 and 1:10 molar ratios in deuterium oxide (Cambridge Isotope Laboratories). Chemical shifts (δ) are in parts per million and internally referenced relative to the solvent nuclei.

Electron paramagnetic resonance (EPR) measurements were performed using a Bruker EMX EPR spectrometer operating at X-band with 100-kHz modulation equipped with EMX standard cavity. Samples of pyocyanin (2 mM), ascorbic acid (10 mM), and pyocyanin:ascorbic acid (1:1) in deuterium oxide were transferred to an EPR capillary and positioned in the EPR cavity; scans were executed promptly. The spectra were recorded using the following instrumental settings: sampling time, 0.00516 s; microwave power, 0.0006325 W; modulation amplitude, 0.0001 T; and modulation frequency, 100000 Hz receiver gain, 30. EPR spectra shown represent the average of 16 scans.

### Electrochemical measurements

DNA sequences (Geneworks) had a C_6_-thiol modification at the 5′ end. Probe: 5′-SH-(CH_2_)_3_-p-TCAACATCAGTCTGATAAGCTA-3′, complementary: 5′-TAGCTTATCAGACTGATGTTGA-3′ and A-C-Mismatch: 5′-TAGCTTATCAAACTGATGTTGA-3′ (mismatch underlined). Components of buffer solutions including potassium phosphate dibasic, potassium phosphate monobasic, sodium chloride, magnesium chloride and 6-mercapto-1-hexanol (C_6_OH) were from Sigma-Aldrich. Prior to modification, the gold disk electrodes (eDAQ, diameter 1 mm) were mechanically polished successively with 1.0, 0.3 and 0.05 mm alumina slurries made from dry Buehler alumina and Milli-Q water on microcloth pads. The final polishing step was performed in the absence of alumina powders. The electrodes were thoroughly rinsed with Milli-Q water after polishing and dried under a stream of N_2_ gas. Electrodes were polished by cycling the potential between −0.3 V and +1.5 V at 0.1 Vs^−1^ in 0.05 M H_2_SO_4_ until a reproducible cyclic voltammogram was obtained followed by washing with Milli-Q water^40^.

Gold electrodes were modified with thiolated probe DNA by incubation of cleaned electrodes in a 1 μM DNA solution in phosphate-buffered saline (PBS) for 2 h. DNA-modified electrodes were thoroughly rinsed with Milli-Q water and treated for 30 min with a 2 mM aqueous solution of C_6_OH, followed by thorough rinsing with Milli-Q water. Hybridization was performed at room temperature by immersing the ssDNA/C_6_OH-modified gold electrodes in 4 μM solutions of target DNA in hybridization buffer (5 mM MgCl_2_ in PBS) for 120 min, followed by rinsing with PBS.

Square wave voltammetry (SWV) measurements were performed in a glass cell using a microAutolab potentiostat (Metrohm, PGSTAT12) equipped with GPES software with an Ag/AgCl (3 M KCl) reference electrode, a platinum wire auxiliary electrode and PBS as the electrolyte. The solution was degassed with argon for at least 15 min before data acquisition. The SWV measurement was performed at pulse amplitude of 25 mV, step of 4 mV and frequency of 10 Hz. The procedure for detection of hybridization by pyocyanin is outlined in Supplementary Figure 2. The ssDNA- and dsDNA-modified electrodes were immersed for 30 min in 100 μM pyocyanin followed by rinsing with 0.5 mL PBS to remove non-specifically bound pyocyanin and transferred into a solution containing 2.5 μM pyocyanin in PBS for the voltametric measurements.

### Viscosity measurements

Viscosities (η) of all samples (pyocyanin, DNA, ascorbic acid, glutathione, DNA-pyocyanin, DNA-pyocyanin-ascorbic acid, DNA-pyocyanin-glutathione, DNA-ascorbic acid and DNA-glutathione) were determined using the rolling ball principle at 20°C under atmospheric pressure using an automated Lovis 2000 ME microviscometer (Anton Paar). A glass capillary (diameter 1.59 mm) with stainless steel roll ball (1.5 mm diameter, specific density 7.70 g/cm^3^) was calibrated and adjusted with degassed water (dynamic viscosity 1.002 mPa·s at 20°C). The viscosity of test samples (0.8 ml) was determined based on equation (3):
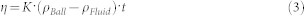
where (η) is dynamic viscosity of the measured sample, *K* is calibration constant specific for each capillary, obtained by calibration with a standard liquid of known viscosity; *ρ_Ball_*and *ρ_Fluid_* are densities of the stainless steel ball and the measured sample, respectively, and *t* is rolling time of the ball between defined marks.

### Generation of biofilm supernatants

*P. aeruginosa* PA14 wild-type and Δ*phzA-G* (phenazine-deficient mutant) strains were plated onto LB agar plates and incubated overnight under aerobic conditions at 37°C. Single colonies from the agar plates were used to inoculate 20-ml cultures in Luria broth (LB) medium (pH 7), with shaking at 150 rpm for 24 h at 30°C. The optical density of both wild-type and Δ*phzA-G* bacterial cultures over 24 h was 2.0 at 600 nm measured using a Bio-Rad Smartspec 3000 (Bio-Rad Laboratories Pvt Ltd, USA) using plain LB medium without bacterial cells as a blank. Cells were harvested and pelleted by centrifugation (4912 × *g*) for 5 min at 10°C and subsequently washed twice and resuspended in PBS (OD = 0.6 ± 0.02). Cells were incubated for 1 h in 6-well Costar polystyrene plates at 30°C and 150 rpm, followed by rinsing with PBS to remove unattached cells. LB (3 ml per well) was then applied to the attached cells and biofilms were grown, shaking at 150 rpm for 24 h at 30°C. After 24 h, the supernatants (biofilm-spent media) of wild-type and Δ*phzA-G* were filtered (0.22 μm Millipore filter) and the viscosity determined. Pyocyanin in supernatants was quantified by absorbance at 691 nm (λ_max_ of pyocyanin)^17^ using a microplate spectrophotometer (VERSA max, Bio-Strategy).

### Biofilm preparation and growth, imaging and analysis

*P. aeruginosa* PA14 wild-type and Δ*phzA-G* mutant strains were plated onto LB agar plates and incubated overnight under aerobic conditions at 37°C. Single colonies were inoculated into 20 ml cultures in LB medium (pH 7) in 100-ml conical flasks and incubated for 24 h at 30°C and 150 rpm. After 24 h of growth, cells were harvested by centrifugation at 5000 × *g* for 5 min at 10°C and resuspended in LB medium to an optical density (600 nm) of 0.5. Biofilms were grown by adding 1.5 ml of bacterial cell suspension into Sarstedt polystyrene petri dishes (35 mm diameter, 10 mm depth) and incubated with lids for 24 h at 30°C and 150 rpm. Where indicated, DNase I (50 U), pyocyanin (200 μM) were included in the incubations. After 24-h incubation, biofilm growth medium was discarded and biofilms were rinsed three times with PBS in order to remove any planktonic/loosely bound bacterial cells. Biofilms were then stained with a live/dead stain (Life Technologies) for 20 min in the dark according to the manufacturer's instructions and visualized by confocal laser scanning microscopy (Olympus FV1200, Australia) with laser settings of 473 nm and 559 nm for green (live) and red (dead) staining, respectively. ImageJ software was employed to generate images and quantify biofilm % surface coverage.

### Statistical analysis

The student's *t*-test was used for statistical analysis and differences were considered significant if *P* < 0.05.

## Author Contributions

T.D., S.K.K., R.T., A.I., J.P., S.S. planned and performed the experiments. L.A., A.W.S.Y., S.R.T., N.K., J.J.G. and M.M. planed the experiments. All authors contributed in writing the manuscript.

## Supplementary Material

Supplementary Information Phenazine virulence factor binding to extracellular DNA is important for Pseudomonas aeruginosa biofilm formation
