# Supplementary material for: Phenazine virulence factor binding to extracellular DNA is important for Pseudomonas aeruginosa biofilm formation
Source: Sci Rep. 2015 Feb 11;5:8398. doi: 10.1038/srep08398 (PMC4323658; doi:10.1038/srep08398)
Supplement: Supplementary Information — Phenazine virulence factor binding to extracellular DNA is important for Pseudomonas aeruginosa biofilm formation [file srep08398-s1.pdf]

Phenazine virulence factor binding to extracellular DNA is important for *Pseudomonas aeruginosa* biofilm formation

Theerthankar Das[1*#] Samuel K. Kutty[2#], Roya Tavallaie[2#], Amaye I. Ibugo[1], Janjira Panchompoo[2], Shama Sehar[1], Leigh Aldous[2], Amanda W. S. Yeung[3], Shane R. Thomas[3], Naresh Kumar[2], Justin J. Gooding[2] and Mike Manefield[1]

[1]School of Biotechnology and Biomolecular Sciences, University of New South Wales (UNSW), Sydney 2052, Australia.

[2]School of Chemistry, University of New South Wales (UNSW), Sydney 2052, Australia.

[3]Centre for Vascular Research and School of Medical Sciences, University of New South Wales (UNSW), Sydney 2052, Australia.

Supplementary Information

Figures

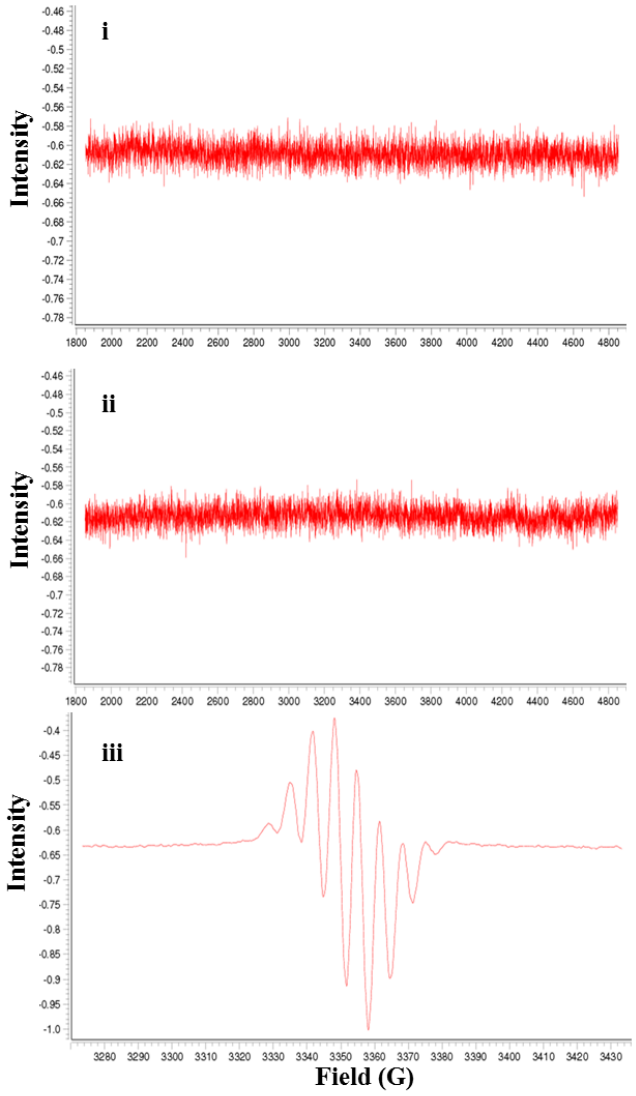

Supplementary figure 1. Reaction between pyocyanin and ascorbic acid.

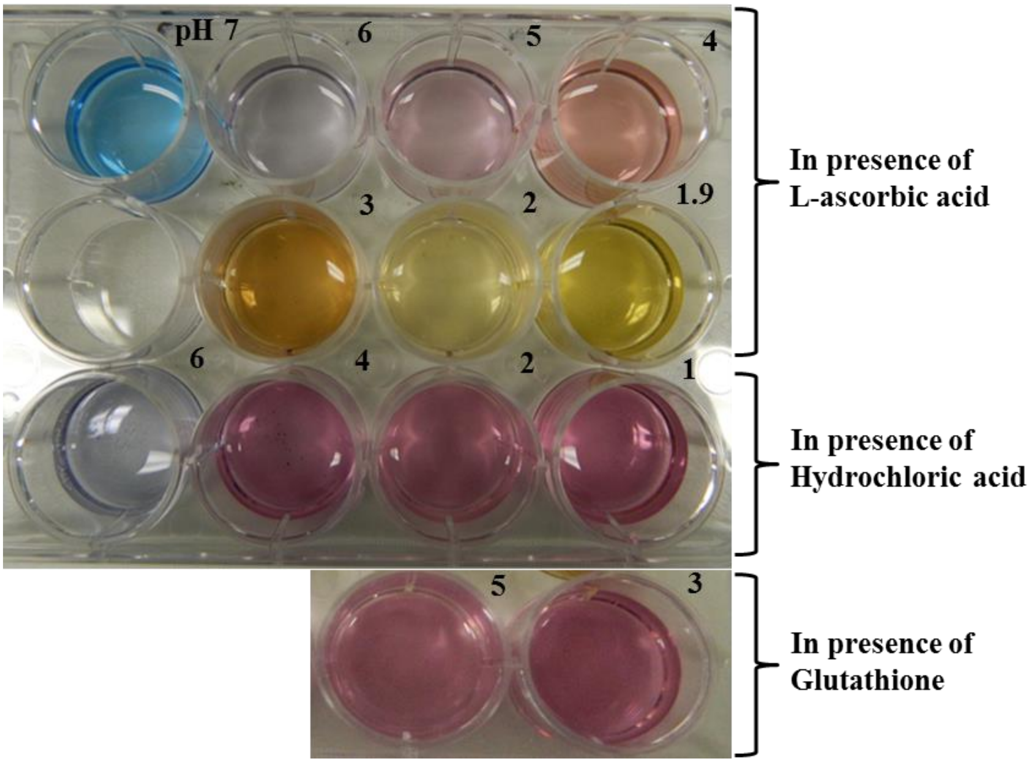

Supplementary figure 2. Differences in reaction between pyocyanin with ascorbic acid or hydrochloric acid or glutathione.

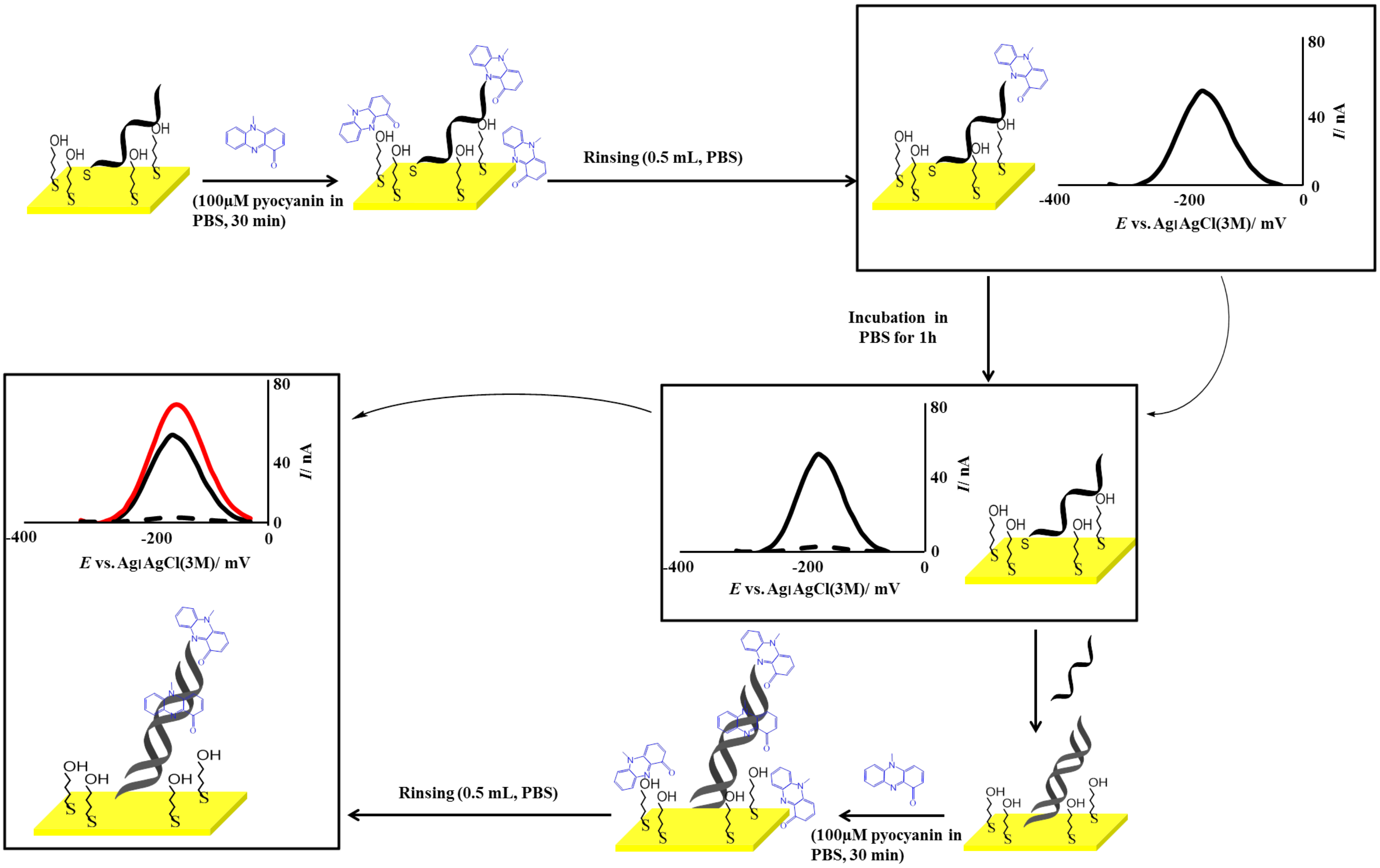

Supplementary figure 3. Fabrication of DNA biosensor using pyocyanin on loosely packed DNA monolayers and transduction of hybridisation event via SWV.

Supplementary figure legends

Supplementary figure 1. Reaction between pyocyanin and ascorbic acid. EPR spectra of i) pyocyanin (2 mM), ii) ascorbic acid (10 mM) and iii) pyocyanin:ascorbic acid (1:1) in deuterium oxide under aerobic condition.

Supplementary figure 2. Differences in reaction between pyocyanin with ascorbic acid or hydrochloric acid or glutathione. Observed change in pyocyanin color from blue to pink, peach and different shades of yellow after reacting with increasing concentrations of ascorbic acid (0-200 mM). This indicates the nature of the charge transfer complex between pyocyanin and ascorbic acid is dependent on the concentration of ascorbic acid. The color of pyocyanin with hydrochloric acid or glutathione (up to 150 mM) remained pink regardless of concentration.

Supplementary figure 3. Fabrication of DNA biosensor using pyocyanin on loosely packed DNA monolayers and transduction of the hybridization event via SWV. The ssDNA and dsDNA-modified electrodes were immersed for 30 min in a solution containing 100 µM pyocyanin in PBS. After accumulation of pyocyanin, the electrode was rinsed with 0.5 mL PBS to remove non-specifically bound pyocyanin and transferred into a solution containing 2.5 µM pyocyanin in PBS for the voltametric measurements. The electrodes were rinsed and incubated for one hour in PBS solution before hybridization to ensure the complete removal of the previously accumulated redox label. This was verified by recording a SWV in the blank buffer solution and checking for the absence of any pyocyanin electrochemistry.
